# Prospects of Levetiracetam as a Neuroprotective Drug Against Status Epilepticus, Traumatic Brain Injury, and Stroke

**DOI:** 10.3389/fneur.2013.00172

**Published:** 2013-11-04

**Authors:** Ashok K. Shetty

**Affiliations:** ^1^Institute for Regenerative Medicine, Texas A&M Health Science Center College of Medicine at Scott & White, Temple, TX, USA; ^2^Research Service, Olin E. Teague Veterans Affairs Medical Center, Central Texas Veterans Health Care System, Temple, TX, USA; ^3^Department of Molecular and Cellular Medicine, Texas A&M Health Science Center College of Medicine, College Station, TX, USA

**Keywords:** traumatic brain injury, stroke, acute seizures, chronic epilepsy, post-traumatic seizures, post-traumatic epilepsy, neurodegeneration, epileptogenesis

## Abstract

Levetiracetam (LEV) is an anti-epileptic drug commonly used for the treatment of partial onset and generalized seizures. In addition to its neuromodulatory and neuroinhibitory effects via its binding to the synaptic vesicle protein SV2A, multiple studies have suggested neuroprotective properties for LEV in both epileptic and non-epileptic conditions. The purpose of this review is to discuss the extent of LEV-mediated protection seen in different neurological conditions, the potential of LEV for easing epileptogenesis, and the possible mechanisms that underlie the protective properties of LEV. LEV has been found to be particularly beneficial for restraining seizures in animal models of spontaneous epilepsy, acute seizures, and status epilepticus (SE). However, its ability for easing epileptogenesis and cognitive dysfunction following SE remains controversial with some studies implying favorable outcomes and others reporting no beneficial effects. Efficacy of LEV as a neuroprotective drug against traumatic brain injury (TBI) has received much attention. While animal studies in TBI models have showed significant neuroprotection and improvements in motor and memory performance with LEV treatment, clinical studies suggest that LEV has similar efficacy as phenytoin in terms of its ability to prevent post-traumatic epilepsy. LEV treatment for TBI is also reported to have fewer adverse effects and monitoring considerations but electroencephalographic recordings suggest the presence of increased seizure tendency. Studies on stroke imply that LEV is a useful alternative to carbamazepine for preventing post-stroke seizures in terms of efficacy and safety. Thus, LEV treatment has promise for restraining SE-, TBI-, or stroke-induced chronic epilepsy. Nevertheless, additional studies are needed to ascertain the most apt dose, timing of intervention, and duration of treatment after the initial precipitating injury and the mechanisms underlying LEV-mediated beneficial effects.

## Introduction

Levetiracetam [LEV; 2*S*-(oxo-1-pyrrolidinyl) butanamide] is an anti-epileptic drug (AED) often utilized for the treatment of partial onset and generalized seizures (1, 2). LEV has both anti-seizure and anti-epileptogenic properties. It has been also proposed that LEV is an attractive AED for managing post-traumatic seizures (PTSs) owing to its beneficial pharmacokinetic attributes, including excellent bioavailability, linear kinetics, minimal plasma protein binding, and rapid achievement of steady state concentrations (2–4). The underlying mechanisms by which LEV facilitates anti-epileptic and anti-epileptogenic effects are different from classic AEDs. Studies insinuate that LEV bestows its effects mainly through the inhibition of the synaptic vesicle protein 2A (1). Additional investigations have also revealed that LEV can inhibit HVA-Ca^2^ channels (N-type), negate the inhibition of negative allosteric modulators such as zinc and β-carbolines of γ-aminobutyric acid (GABA)- and glycine-gated currents, and diminish the calcium release from intraneuronal stores (1, 5).

Moreover, a multitude of studies have proposed that LEV has considerable neuroprotective properties in both epileptic and non-epileptic disorders (2, 6–9). The capability of LEV to augment the manifestation of glial glutamate transporters EAAT1/GLAST and EAAT2/GLT-1 has been proposed as one of the foremost mechanisms through which LEV mediates its neuroprotective properties (2, 10). This hypothesis fits well with one of the conspicuous changes detected following most brain insults, which is increased concentration of glutamate in the extracellular areas causing enhanced activation of *N*-methyl-d-aspartate (NMDA) receptors and α-amino-3-hydroxy-5-methyl-4-isoxazolepropionic acid (AMPA) receptors on neurons and culminating in significant neurodegeneration (2, 11, 12). The efficacy of LEV as a neuroprotective compound has been examined in several brain injury and neurodegenerative disease prototypes. These include brain damage resulting from status epilepticus (SE) or acute seizures, spontaneous epilepsy, closed head trauma, subarachnoid hemorrhage (SAH), hypoxic-ischemia, and stroke. The goal of this review is to confer the extent of LEV-mediated neuroprotection observed in different brain injury models, the potential of LEV for easing epileptogenesis, and the possible mechanisms that underlie neuroprotective properties of LEV in different neurological conditions.

## Efficacy of LEV for Easing Seizures and Seizure-Mediated Neurodegeneration

Levetiracetam administration appears to be beneficial for restraining seizures, and acute seizure or SE induced neurodegeneration in animal models. A single dose of LEV administered 30 min after the onset of behavioral SE was adequate for transiently attenuating seizure activity in animals treated with LEV at 800 mg/kg or higher (13). Increased doses of LEV (1000 mg/kg or higher) dampened behavioral seizures for prolonged periods. When administered early (i.e., 10 min) after the onset of SE, 400 mg/kg of LEV transiently attenuated behavioral seizures and higher doses dampened seizures for relatively longer periods. Pretreatment with LEV prior to pilocarpine injection delayed the onset of seizures but did not significantly alter ictal discharge measured through electroencephalographic (EEG) recordings (13). Analyses with TUNEL staining however demonstrated reduced neuronal injury in the hippocampus and other limbic brain regions in animals that responded behaviorally to LEV.

Levetiracetam treatment appears to mediate anti-seizure effects through several mechanisms. A study using acute hippocampal slices from spontaneously epileptic rats (SERs) have suggested that LEV modulates Ca^2+^ currents in neurons, as application of 10 μM of LEV decreased the amplitude of the Ca^2+^ current in CA3 pyramidal neurons and application of 100 nM–1 mM of LEV reduced the Ca^2+^ current in a concentration-dependent manner (14). LEV also elevated the threshold potential level for activation of the Ca^2+^ current and reduced the L-type Ca^2+^ current in neurons. Furthermore, LEV can abolish the SE-induced rise in brain-derived neurotrophic factor, a neurotrophic factor believed to contribute to seizures at higher concentrations. Moreover, administration of LEV after SE can enhance levels of Y1- and Y5-like receptors of neuropeptide Y (NPY; an endogenous anticonvulsant) in all subfields of the hippocampus (15). Also, anti-epileptic effect of LEV is apparent from a study in an animal model of hypoxia-induced seizures (16). LEV pretreatment in postnatal day 10 rats significantly decreased the cumulative duration of hypoxia-induced behavioral and electrographic seizures at 25 and 50 mg/kg doses. Additionally, kainate-induced seizures and neuronal loss were significantly diminished in postnatal day 40 rats previously treated with LEV. Thus, LEV treatment can not only suppress acute seizures but also diminish later-life seizure susceptibility and seizure-induced neuronal injury. This suggests that LEV treatment after injury or acute seizures has potential for disease modification.

A recent study has examined the neuroprotective property of LEV against SE in greater detail (17) by administering the drug 2 h after the onset of SE at 50, 100, or 150 mg/kg. Analyses through staining for Fluoro-Jade B (a marker of degenerating neurons) suggested that low dose administration of LEV (50 mg/kg) can reduce SE-induced loss of CA1 pyramidal neurons and dentate hilar neurons but not CA3 pyramidal neurons. However, LEV treatment at a higher dose (100 mg/kg) reduced degenerating neurons in CA1 and CA3 pyramidal cell layers as well as the dentate hilus. Furthermore, a much higher dose of LEV (150 mg/kg) greatly reduced the numbers of degenerating neurons in the CA3 pyramidal cell layer. Interestingly, when different doses of LEV were combined with diazepam (10 mg/kg), neurodegeneration was exacerbated in the hippocampus. Collectively, this study demonstrated that LEV alone is more efficacious for preventing SE-induced neurodegeneration in the hippocampus than other AEDs such as diazepam or valproate (17). Furthermore, the finding that combined administration of LEV and diazepam actually increases neurodegeneration suggested that LEV negatively interacts with diazepam, implying that LEV may be more suitable as a first line drug to minimize SE-induced neurodegeneration rather than as an add-on drug with benzodiazepines (17). Thus, LEV administration after the onset of SE is beneficial for suppressing seizures as well as reducing neurodegeneration. Mechanisms of LEV-mediated neuroprotection likely include its anti-seizure effects as well as its purported ability to decrease the expression of pro-oxidant protein iNOS and increase the expression of the antioxidant protein cystine/glutamate exchanger in the hippocampus (18). A study in pilocarpine model of SE also showed that LEV pretreatment could counteract oxidative stress through maintenance of lipid peroxidation, nitrite-nitrate levels, catalase activity, and glutathione at normal levels in the hippocampus (19).

## Usefulness of LEV for Easing Epileptogenesis

Prolonged LEV treatment after SE appears to delay or restrain the development of chronic epilepsy in animal models. A study examined the effects of chronic LEV treatment on hippocampal field responses in rats subjected to pilocarpine induced SE (20). Hippocampal field potentials were recorded *in vivo* in anesthetized animals after 3-day washout period that followed 21-day treatment with different doses of LEV (50, 150, or 300 mg/kg/day) administered via osmotic minipumps. Chronic treatment with LEV yielded clinically relevant plasma concentrations throughout the experiment with complete washout of the drug 3 days after treatment cessation. At this point of time post-SE rats chronically treated with vehicle developed clear signs of hippocampal hyperexcitability typified by increased amplitude of population spike (PS) recorded in the DG and reduced paired-pulse inhibition in the CA1 area. LEV treatment dose-dependently counteracted these long-term effects of SE. Furthermore, at the dose of 300 mg/kg/day, LEV restored these parameters back to control levels (20). Several other studies have also shown beneficial effects of LEV treatment in acute seizure models. For example, the development of kindling (a progressive increase in seizure severity induced by repeated brain stimulation at certain intervals), and kindling-related abnormal gene expression can be considerably modulated through daily application of LEV (15, 21, 22).

Furthermore, LEV administration at 40 mg/kg is efficacious not only for suppressing the development of kindling but also for dampening kindling-induced expression of multiple immediate early genes (IEGs) including many synaptic plasticity-related IEGs, and some late response genes encoding transcription factors, neurotrophic factors, and proteins that are known to regulate synaptic remodeling (23). An additional potential mechanism by which LEV suppresses the development of kindling is through significant inhibition of kindling-induced synaptic potentiation (24). LEV treatment after kindling can also prevent asymmetric accumulation of hippocampal 7S SNARE complexes [the secretory machinery responsible for neurotransmitter (NT) release] and accumulation of SV2 (25). Thus, LEV treatment can ease multiple abnormalities induced by kindling at cellular and molecular levels.

Moreover, a study in a kainate model of SE examining the long-term effects of LEV treatment, commencing a day after the onset of SE and continuing for 25 days (26) demonstrated that LEV treatment after SE can decrease the mean duration of spontaneous electrographic seizures in the chronic phase after SE. Interestingly, LEV administration also greatly eased SE-induced aberrant migration of newly born neurons into the dentate hilus, an abnormal process that is believed to contribute to the formation of aberrant hippocampal circuitry and epileptogenesis after SE (27, 28). LEV administration has also been found to be effective for easing inflammatory responses in the hippocampus and piriform cortex of epileptic rats (29). These brain regions in epileptic animals typically demonstrate reactive astrocytes and activated microglia displaying strong expression of IL-1β and interleukin-1 receptor subtype 1 (IL-1R1). Interestingly, LEV administration reduced reactive gliosis and expression levels of IL-1β in both of these brain regions. These findings suggested that LEV likely mediates its anti-epileptogenic effects at least partially through modulation of inflammation in epileptic brain regions. Studies in SERs have also suggested anti-epileptogenic effects of LEV (30). Administration of LEV (80 mg/kg/day) to SERs from postnatal weeks 5–8 significantly inhibited seizures at postnatal weeks 5–13. It is of interest to note that inhibition of seizure expression in SERs was still apparent 5 weeks after the termination of LEV treatment, reinforcing that LEV possesses anti-epileptogenic properties.

From the above studies, it is tempting to conclude that chronic treatment with LEV is efficacious for restraining the evolution of initial SE-induced brain insults into a state of hippocampal hyperexcitability and chronic epilepsy. However, currently, there is no clear consensus regarding anti-epileptogenic effects of LEV in SE models. For instance, a study in amygdala kindling model of SE showed that prophylactic treatment with LEV has no effect on epileptogenesis, neuronal damage, or behavioral alterations in rats (31). In one set of studies, LEV treatment was initiated 24 h after onset of electrical amygdala stimulation without termination of SE and continued for 8 weeks using osmotic minipumps. In another set of studies, LEV treatment commenced 4 h after the onset of SE with seizure termination through diazepam and continued for 5 weeks. Interestingly, with either treatment regimen, LEV did not exert anti-epileptogenic or neuroprotective activity. Furthermore, behavioral hyperexcitability and learning deficits were not affected by treatment with LEV after SE. Another study investigating the effects of LEV on visual-spatial memory following SE corroborated these findings (32). Adult rats subjected to SE were treated first with LEV or vehicle for 14 days, tested for visual-spatial memory in the Morris water-maze and then used for unit recording in the CA1 region of the hippocampus. Animals undergoing SE displayed impaired learning and memory function in the water-maze test and abnormalities in firing patterns of pyramidal neurons (place cells) in the CA1 cell layer. LEV treatment had no major effects on water-maze performance or place cell function. Histological analyses however revealed severe neurodegeneration in the CA1 pyramidal cell layer of rats receiving vehicle after SE and relatively reduced neurodegeneration in rats receiving LEV after SE.

Thus, the extent of neuroprotection mediated by LEV treatment was not adequate for preventing SE-induced cognitive dysfunction. However, discrepancy in results between studies may reflect differences in species and strains of animals examined, timing, and dose of LEV treatment after SE, and severity of SE at the time of commencement of LEV treatment. Timing of treatment after SE is particularly important because a study using a rat perforant pathway stimulation model has shown that administration of LEV 5 h after SE does not protect from mitochondrial dysfunction but LEV treatment during established SE prevents mitochondrial dysfunction (7, 33).

## Promise of LEV for Mediating Neuroprotection Against Traumatic Brain Injury

Investigation of the effects of LEV in animal models of closed head injury (CHI) and SAH suggested that LEV is neuroprotective against traumatic brain injury (TBI) (6). In this study, a single intravenous dose of LEV has been shown to improve both functional and histological outcomes after CHI. Moreover, the beneficial effects seemed specific for LEV treatment as fosphenytoin administration did not result in such effects. Administration of LEV also improved functional outcomes and reduced vasospasm following SAH. This was the first study to suggest that LEV could be a therapeutic alternative to phenytoin for TBI in clinical situations where seizure prophylaxis drugs are indicated. Moreover, a recent study has examined the effects of LEV on motor and cognitive function in a rat prototype of TBI (2). Adult male rats were administered LEV (50 mg/kg, i.p) or vehicle daily for 20 days beginning 1 day following a controlled cortical impact (CCI) injury or sham surgery. Animals were assessed for various behavioral tests, which comprised assessment of motor function via beam walking test and spatial learning and memory function through Y-maze and Morris water-maze tests. The results showed that daily LEV treatment for 20 days improved motor function and enhanced novel arm exploration in the Y-maze. Furthermore, LEV treatment promoted greater sparing of hippocampal neurons, decreased contusion volumes, reversed TBI-induced decreases observed in glutamate transporters and markers that promote neuroplasticity, and reduced the expression of pro-inflammatory cytokine IL-1β. However, LEV treatment did not improve spatial learning ability in rats with TBI. Collectively, these animal studies imply that daily LEV treatment has favorable effects on structural, molecular, and some of the behavioral components of neurological improvements after TBI, likely through modulation of excitatory and neuroinflammatory pathways (2).

Additionally, several clinical studies have ascertained the efficacy of LEV for preventing PTS or post-traumatic epilepsy (PTE). Amongst ∼275,000 individuals who are typically hospitalized with TBI every year, ∼7% experience PTS (34). As per guidelines of the Brain Trauma Foundation and the American Academy of Neurology for the management of severe TBI, administration of AEDs to prevent PTS is recommended only through the initial 7 days after TBI (34). Amid AEDs, the efficiency of phenytoin treatment has been extensively examined for preventing PTS after TBI. However, several clinical studies suggest that LEV treatment after TBI is also effective for decreasing the predilection for developing PTS. Jones and colleagues analyzed EEG recordings from patients receiving phenytoin or LEV for seizure prevention following severe TBI (35). This comparative analysis revealed that LEV is as efficient as phenytoin in averting early PTS but is allied with an increased seizure predisposition based on evaluation of EEG recordings. Another open label, non-randomized phase 2 study assessed the safety, tolerability, and effectiveness of LEV therapy in patients with TBI exhibiting greater susceptibility for PTE (36). LEV treatment was initiated within 8 h after injury and continued for 30 days in this study. Two-year follow-up uncovered that occurrence of PTE in patients receiving LEV (11%) is less than that observed in untreated TBI patients (20%). However, several recent clinical studies in TBI patients report that LEV does not outperform phenytoin as a prophylaxis drug against PTS (37, 38). Another recent clinical study has reported that LEV treatment to children ages 6–17 years with risk factors for the development of PTE decreased the incidence of PTE, as only 1 in 40 patients receiving LEV displayed PTE (39). Collectively, from the above studies, it emerges that LEV has analogous ability as phenytoin for thwarting PTS after TBI. It is also reported that LEV treatment for TBI is linked with fewer adverse effects and monitoring considerations [for details, see the review by Ref. (34)]. Nonetheless, because LEV administration was accompanied by an increased seizure propensity (35), the Brain Trauma Foundation has recommended using phenytoin for early PTS prophylaxis (34).

## LEV as a Neuroprotective Compound Against Stroke

Seizures following stroke is one of the causes of epilepsy in adults, particularly in elderly patients (40). Seizures typically occur in ∼10% of stroke patients, depending on risk factors, such as the type of stroke, location of stroke-induced damage in the brain, and severity of the stroke (40). However, stroke accounts for ∼50% of seizures in individuals above the age of 65 years (41). Classically, the use of AEDs to avert recurrent post-stroke seizures is recommended. LEV has been suggested as a first-choice drug against post-stroke seizures, based on safety and efficacy profiles in clinical studies (42). Kutlu and colleagues examined the suitability of LEV monotherapy in individuals aged 60 or older and exhibiting a minimum of two late-onset post-stroke seizures (43). At daily doses of 1000–2000 mg, they reported that 82.4% of the patients were seizure free but seven patients (20.6%) had side effects. These results suggested that LEV monotherapy is efficient and well tolerated in elderly patients with late-onset post-stroke seizures. Consoli and associates compared the efficacy of LEV treatment with carbamazepine (CBZ) in patients with post-stroke seizures in a multicenter randomized open label study (41). Evaluation of results in 106 patients (52 treated with LEV and 54 treated with CBZ) showed no noteworthy variance in the number of seizure free patients between LEV and CBZ. Yet, interval to the first recurrence tended to be longer in patients receiving LEV. The results also suggested that LEV treatment caused considerably less side effects than CBZ, as attention deficit, frontal executive functions, and functional scales (Activities of Daily Living and Instrumental Activities of Daily Living indices) were notably poorer in patients receiving CBZ (41).

Thus, studies conducted so far imply that LEV is a useful alternative to CBZ for post-stroke seizures, predominantly in terms of efficacy and decreased adverse effects. However, another recent study reported that LEV is not effective for the treatment of central post-stroke pain, a severe chronic neuropathic pain state called allodynia resulting from a vascular lesion (44). Considering these, further studies are needed to ascertain the efficacy of LEV as a suitable neuroprotective and seizure-preventing drug after stroke. Additionally, rigorous studies in animal models of stroke are needed to understand the potential anti-seizure, neuroprotective, and anti-epileptogenic effects of LEV following stroke.

## Overall Conclusion

From the analysis of literature pertaining to LEV treatment mediated protection in neurological disorders, it emerges that LEV treatment has potential for restraining SE-, TBI-, and stroke-induced chronic epilepsy development. Particularly, LEV administration has been found to be advantageous for restraining seizures and/or seizure-induced neurodegeneration in animal models of spontaneous epilepsy, acute seizures, and SE. LEV treatment appears to mediate anti-seizure and neuroprotective effects via modulation of Ca^2+^ currents in neurons, inhibition of the up-regulation of brain-derived neurotrophic factor, increases in NPY receptors, and antioxidant proteins, and decreases in pro-oxidant proteins (14, 15, 17). Nonetheless, the capability of LEV for easing epileptogenesis and cognitive dysfunction following SE remains contentious. Several studies report promising outcomes such as delayed development of hippocampal hyperexcitability, restraint of electrographic seizures, diminishment in the abnormal migration of newly born neurons into the dentate hilus, and inhibition of inflammatory responses in SE models (20, 26, 27, 29). There are also reports of mitigation of synaptic potentiation and abnormal expression of IEGs, and prevention of abnormal accumulation of 7S SNARE complexes and SV2 in kindling models (15, 22–24). However, some studies report no beneficial effects of LEV treatment after SE in terms of easing epileptogenesis or cognitive dysfunction (31, 32). Discrepancy in the findings between studies may reflect differences in the timing of intervention with LEV, doses of LEV employed and severity of SE at the time of initial intervention with LEV.

The efficiency of LEV as a neuroprotective drug against TBI has received much consideration. Animal studies in TBI models validate greater sparing of hippocampal neurons, and improved motor and memory function with LEV treatment (2, 6). On the other hand, results of several recent clinical trials convey that LEV has comparable efficacy as phenytoin in terms of its ability for preventing PTE (34, 37–39). The other positive effects of LEV treatment for TBI comprise fewer adverse effects and monitoring issues. However, one caveat of LEV treatment for TBI is the presence of increased seizure propensity in long-term EEG recordings (34, 35). Studies on stroke imply that LEV is a useful alternative to CBZ for preventing post-stroke seizures including elderly patients, in terms of efficacy and safety (40–43) but does not seem to be useful for easing central post-stroke pain (44). Taken together, studies conducted so far suggest that LEV treatment is useful for easing SE-, TBI-, and stroke-induced chronic epilepsy development. Nevertheless, rigorous additional studies in animal models are needed to ascertain the most beneficial dose, timing of intervention, and duration of treatment after the initial precipitating injury and mechanisms underlying LEV-mediated beneficial effects on epileptogenesis.

## Conflict of Interest Statement

The author declares that the research was conducted in the absence of any commercial or financial relationships that could be construed as a potential conflict of interest.
